# Implicit attitudes and explicit cognitions jointly predict a reduced red meat intake: a three-wave longitudinal study

**DOI:** 10.1080/21642850.2020.1730843

**Published:** 2020-02-23

**Authors:** Carolin Muschalik, Rik Crutzen, Math J. J. M. Candel, Iman Elfeddali, Hein de Vries

**Affiliations:** a Department of Health Promotion, Care and Public Health Research Institute (Caphri), Maastricht University, Maastricht, Netherlands; b Department of Methodology and Statistics, Care and Pubic Health Research Institute (Caphri), Maastricht University, Maastricht, Netherlands; c GGz Breburg, Academic Department of Specialized Mental Health Care, Tilburg, Netherlands; d Tilburg University, Tranzo – Scientific Center for Care and Welfare, Tilburg, Netherlands

**Keywords:** Red meat consumption, intention, implicit attitudes, explicit cognitions, interactions

## Abstract

**Background:** Despite nutritional benefits, a high consumption of red meat is not without risks as it is linked to the development of certain types of cancer as well as to other non-communicable diseases, such as type II diabetes or cardiovascular diseases. Moreover, the production of meat has negative effects on the environment. Therefore, a transition to a less meat-based diet could be beneficial. It is unclear how explicit cognitions towards red meat consumption and implicit attitudes jointly influence intention and consumption. We tested the additive pattern (both types of cognitions explain unique variance) and interactive pattern (both types interact in the prediction).

**Method:** At baseline (T0; *N* = 1790) and one (T1; *n* = 980) and three months thereafter (T2; *n* = 556), explicit cognitions, red meat consumption, and implicit attitudes were assessed among a Dutch sample.

**Results:** Only explicit cognitions were associated with red meat consumption. Implicit attitudes moderated the effect of self-efficacy on T0-RMC; negative implicit attitudes strengthened this effect. T0-intention was associated with explicit cognitions and implicit attitudes. Additionally, negative implicit attitudes strengthened the effect of social norms on T0 and T2-intention. Regarding red meat consumption, support for the interactive pattern was found. For intention there was support for the interactive and additive pattern.

**Conclusion:** Interventions aiming to reduce red meat consumption in the general public might profit from changing implicit attitudes in addition to explicit cognitions.

## Introduction

Red meat can be an important dietary source of protein and essential nutrients, such as iron, zinc and vitamin B12 (Chan, McCance, & Brown, 1996; Johnston, Prynne, Stephen, & Wadsworth, 2007). However, when consumed excessively, it can also be a threat to people’s health as it is associated with the development of several diseases, e.g. colon and lung cancer (Cross et al., 2007; Giovannucci et al., 1994), cardiovascular diseases (Kelemen, Kushi, Jacobs, & Cerhan, 2005; Kontogianni, Panagiotakos, Pitsavos, Chrysohoou, & Stefanadis, 2008; Sinha, Cross, Graubard, Leitzmann, & Schatzkin, 2009), type II diabetes (Pan et al., 2011; Song, Manson, Buring, & Liu, 2004), obesity (Pan et al., 2012), and increased mortality in general (Sinha et al., 2009). In 2015, this led the World Health Organization (WHO) to classify the consumption of processed red meat – referring to meat preserved by smoking, curing or salting, or the addition of chemical preservatives, including that contained in processed foods – as carcinogenic and the consumption of red meat – speaking of beef, pork, lamb, and goat from domesticated animals including that contained in processed foods – as potentially carcinogenic (Bouvard et al., 2015; McGuire, 2016). The individual recommendation for people who eat red meat is to reduce their intake to no more than 500 g a week of red meat (equiv. 26 kg/year) and very little if any processed red meat (Adams et al., 2005; World Cancer Research Fund/American Institute for Cancer Research, 2018). The public health goal for the population average consumption of red meat should be no more than 300 g a week (equiv. 14.4 kg/year) and very little if any of which to be processed (Marmot et al., 2007). With an annual average consumption of 43.4 kg per person, European citizens exceed this recommendation by far (Chemnitz & Becheva, 2014). Dutch citizens consume far more red meat than recommended: in 2017 they ate on average 54.6 kg of red and processed red meat per person and year, which does not even take into account the amount of red meat entailed in ready-made meals. Furthermore, meat consumption does not only have negative effects on people’s health, but also on the health of animals and the planet (Aiking, 2014; Friel et al., 2009; Steinfeld, Gerber, Wassenaar, Castel, & De Haan, 2006; Westhoek et al., 2014). Hence, a transition to a less meat-based diet could be beneficial (Scott, 2017). As a natural decrease in red meat intake is not expected in the near future (Chemnitz & Becheva, 2014), a deeper understanding of the cognitions that determine a moderate red meat intake is needed in order to inform future intervention efforts.

In order to explain health behaviors, two approaches can be chosen which are not necessarily mutually exclusive. Social cognitive models, which are the more traditional approach, focus on explicit, deliberate, and volitional constructs. These models explain an individual's intention and behavior by (beliefs underlying) determinants that people can reflect on and can express consciously (i.e. they are reasoned, but not necessarily rational). These determinants are, for example, explicit attitude, self-efficacy or social norms. Another approach focuses on unconscious and more automatic associations that are less subject to reflection by an individual, meaning that they occur regardless of whether the individual perceives them as valid or invalid (Gawronski & Bodenhausen, 2006). These associations are called implicit associations^1^ and are assumed to have one or several of the following properties: unconscious, uncontrollable, efficient, and involuntary (Greenwald & Banaji, 1995). As human behavior is probably neither solely consciously nor unconsciously regulated (Vrabel & Zeigler-Hill, 2017), we argue that in order to understand the consumption of red meat, it is important to take both conscious and unconscious constructs into account. Until now, constructs derived from both approaches have been examined in a few studies on meat intake, however, as far as we know only in isolation from each other (Barnes-Holmes, Murtagh, Barnes-Holmes, & Stewart, 2010; Berndsen & van der Pligt, 2005; Carfora, Caso, & Conner, 2017a; De Houwer & De Bruycker, 2007; Graça, Calheiros, & Oliveira, 2015; Lea & Worsley, 2001). The aim of the study at hand is therefore to investigate how conscious and unconscious constructs together predict a (reduced) red meat intake.

According to social cognitive models, such as the Reasoned Action Approach (Fishbein & Ajzen, 2011) or the I-Change model which integrates constructs from various social cognitive models (De Vries, 2017; De Vries, Mesters, Van de Steeg, & Honing, 2005), an individual’s intention is the most proximal cause for behavior. Intention in turn is formed by three key determinants: (1) a person’s explicit attitude towards the behavior (comprised of perceived pros and perceived cons, i.e. the perceived advantages and disadvantages the behavior entails), (2) social influence (comprised of social modeling and social norms, i.e. how people in one’s environment behave and the perceived social pressure to perform a behavior) and (3) self-efficacy (i.e. the perceived ability or difficulty of performing the given behavior). These constructs were indeed strongly associated with reduced (red) meat intake. That is, positive explicit attitudes towards reducing red meat consumption increased the intention to reduce one’s intake (Carfora, Caso, & Conner, 2017a), whereas positive explicit attitudes towards meat decreased the intention (Graça et al., 2015). Moreover perceiving oneself as capable of changing one’s meat consumption (i.e. self-efficacy) predicted the intention to reduce one’s consumption (Carfora, Caso, & Conner, 2017a; Graça et al., 2015) and a higher number of vegetarian friends (social modeling) is inversely related to an individual’s red meat consumption (Lea & Worsley, 2001). A high intention, in turn, has been shown to result in a lower actual intake of meat (Berndsen & van der Pligt, 2005; Carfora, Caso, & Conner, 2017a).

The contemporary approach of combining implicit and explicit cognitions as determinants of behavior is depicted in dual process models (Hofmann, Friese, & Wiers, 2008; Sheeran, Gollwitzer, & Bargh, 2013; Strack & Deutsch, 2004; Wilson, Lindsey, & Schooler, 2000). The Reflexive-Impulsive Model (RIM) (Strack & Deutsch, 2004) is one example of a dual-process model, which distinguishes between a reflective and an impulsive system. The impulsive system is defined as composition of automatic behavioral tendencies and the reflective system is understood as the composition of reasoned, deliberate, and conscious motives to pursue a behavior. According to Perugini (2005) there exist three possible ways in which the impulsive and reflexive system can operate in guiding a behavior: (1) both systems explain unique variance in the behavior (*additive pattern*), (2) the impulsive system predicts spontaneous and the reflective system predicts deliberate behavior but not vice versa (*double dissociation pattern*) and (3) both types of systems interact synergistically in the prediction of behavior (*interactive pattern*), i.e. one type of determinant strengthens or weakens the effect of the other type of determinant on behavior. Implicit attitudes are one type of implicit (or impulsive) processes that are activated automatically and occur partially or completely outside a person’s awareness (Gawronski & Bodenhausen, 2014; Rydell & McConnell, 2006).

Implicit attitudes are clearly distinguished from the abovementioned explicit attitudes that are incorporated in the social-cognitive models and are commonly assessed by means of self-reported questionnaires. Implicit attitudes, on the contrary, are inferred by computerized reaction time tasks, of which the Implicit Association Test (IAT) (Greenwald, McGhee, & Schwartz, 1998) is the most used one. Briefly, the IAT measures the relative strength of attitudes towards opposing targets (e.g. men vs. women or black vs. white). In order to measure absolute attitudinal strength (e.g. when the question involves predicting responses to one specific target or when a natural opposing target is not identifiable), the Single-Category Implicit Associations Task (SC-IAT) (Karpinski & Steinman, 2006) was designed. Contrary to the IAT, it requires only one target concept (e.g. meat), and one target attribute (e.g. negative) and a contrast attribute (e.g. positive), which are represented by stimuli that participants have to sort as quickly as possible to given categories. The idea underlying this method is that respondents are more likely to react quickly when the concept and the attribute are closely associated in memory (e.g. meat and positive) and more slowly when the concept and the attribute are not or less associated with each other (e.g. meat and negative). Based on the performance, the participant’s implicit attitude is inferred. Implicit attitudes are correlated with food intake in general (Conner, Perugini, O'Gorman, Ayres, & Prestwich, 2007; Friese, Hofmann, & Wänke, 2008; Maison, Greenwald, & Bruin, 2001). Also as in the context of (red) meat consumption, implicit attitudes have been demonstrated to be crucial. That is, meat eaters showed a less positive implicit attitude for vegetables relative to meat compared to vegetarians (De Houwer & De Bruycker, 2007) and they also indicated a small pro-meat tendency compared to vegetarians (Barnes-Holmes et al., 2010).

Previous work has demonstrated that both implicit and explicit cognitions play a role in (reduced) red meat intake, but studies focusing on the mode of operation between them resulted, depending on the target behavior, in mixed findings. A study on snack versus fruit intake supported the double dissociation pattern and a study with smoking as target behavior supported the interactive pattern (Perugini, 2005). Another study on the behavioral choice of fruits or snacks found support for the additive pattern (Richetin, Perugini, Prestwich, & O'Gorman, 2007) and studies regarding physical activity found support for the additive pattern (Bluemke, Brand, Schweizer, & Kahlert, 2010; Calitri, Lowe, Eves, & Bennett, 2009; Conroy, Hyde, Doerksen, & Ribeiro, 2010) as well as for the interactive pattern (Cheval, Sarrazin, Isoard-Gautheur, Radel, & Friese, 2015; Muschalik, Elfeddali, Candel, & De Vries 2018; Perugini, 2005). These findings do not only demonstrate that the mode of operation does not only depend on the target behavior but also that implicit and explicit cognitions can regulate one and the same behavior in accordance with an additive *and* an interactive pattern. This seems logical as both patterns do not necessarily exclude each other: implicit attitudes and explicit determinants could have a direct effect on behavior (suggesting an *additive pattern*) and also interact with each other (suggesting an *interactive pattern*). Therefore, we expect that also in the context of red meat consumption, behavior can be directly influenced by both types of cognitions (*additive pattern*) and that both types of cognitions can interact and either reinforce or weaken each other (*interactive pattern*).

Additionally, we argue that it is important to not only shed light on behavior but also on the intention to reduce one’s red meat intake. As displayed in the social-cognitive models, intention is understood as the most proximate determinant for behavior and thereby an important prerequisite for behavioral purposes. Hence, understanding the process of intention formation would be a first step in the direction of behavioral change. In a study of Muschalik et al. (2018), implicit attitudes moderated the effect of certain explicit cognitions (e.g. perceived pros, social modeling, self-efficacy) on the intention to be physically active and we expect these results to be transferrable to the context of a reduced red meat intake. That is, the positive effect of perceived pros on the intention to reduce one’s intake is expected to be reinforced by negative implicit attitudes towards red meat whereas positive implicit attitudes towards red meat are assumed to weaken the same relation. The same reasoning could be applied to other predictors of intention such as self-efficacy for instance: the positive effect of self-efficacy on intention could be reinforced by negative implicit attitudes towards red meat but weakened by positive implicit attitudes. Furthermore, a few studies have investigated the direct effect of implicit processes or attitudes, respectively, on intention. For example, Cin et al. (2007) revealed that after exposure to a movie in which the protagonist smoked, self-smoking associations as measured by the IAT, were increased which in turn predicted changes in the intention to smoke. Additionally, von Hippel, Brener, and von Hippel (2008) provided evidence that nurses’ implicitly measured prejudice toward injecting drug users predicted unique variance in the behavioral intention to change jobs. Brochu and Morrison (2007) showed that implicit weight biases predicted participants’ behavioral intentions to interact socially with an overweight male person. Based on the findings that implicit processes, such as implicit attitudes, have a direct effect on intention besides explicit predictors, we expect that implicit attitudes also have a direct effect on the intention to reduce red meat intake besides explicit cognitions and will therefore investigate this hypothesis in addition. Given the fact that a single behavior was considered, the double dissociation pattern could not be tested.

Hence, the aim of the study was fourfold. First, we investigated whether implicit attitudes and explicit cognitions predict (a reduced) red meat intake separately from each other (*additive pattern*). Secondly, we explored if implicit attitudes and explicit cognitions interact in the prediction of (a reduced) red meat consumption (*interactive pattern*). Third, we assessed if implicit attitudes interact with explicit cognitions (perceived pros, perceived cons, social norms, social modeling, self-efficacy) in the short and long-term prediction of the intention to reduce one’s red meat intake (*interactive pattern*) and fourth, if implicit attitudes also have a direct effect on intention in the short-term as well as long-term (*additive pattern*).

## Method

### Design

A three-wave longitudinal study was conducted with a baseline measurement (T0), a follow-up after one month (T1) and another follow-up after three months (T2). We have preregistered the study protocol at https://osf.io/vrdqw/register/565fb3678c5e4a66b5582f67?view_only=bc77614ed5154078b43cf9474aa2a9c3 before data collection. Furthermore, materials used in this study as well as non-identifiable data, syntax, and output of the analyses are available at https://osf.io/7enj9/. These efforts are taken to acknowledge a call for full disclosure to maximize scrutiny, foster accurate replication, and facilitate future data syntheses (e.g. meta-analyses) (Peters, Abraham, & Crutzen, 2012).

### Ethical approval

Ethical approval was obtained from the FHMLRec at Maastricht University, the Netherlands (Muschalik/220517).

### Power analysis

To determine the sample size, a power analysis was conducted using G*Power. For the sample calculation it was assumed that the full regression model would explain 7.5% of the variation in the outcome. The contribution of the interaction term to *R*-square was estimated as 3%. Hence, we anticipated a small effect size (*f*2 = 0.03242) for a main effect or interaction effect of implicit attitude and set the test power at 0.80 with a type I error rate of *α* = 0.05 for two-sided testing. These numbers were used in G*power, which revealed a sample size of *N* = 244. Since the predictors in the regression model were likely to be correlated with each other, a correction needed to be done. We assumed that 50% of the variation in the interaction term can be explained by the other predictors of the regression model, leading to a Variance Inflation Factor, VIF = 1/(1–0.5) = 2. For sufficient power, *N* needs to be multiplied with this VIF (Hsieh, Bloch, & Larsen, 1998). Hence, *N* = 2 * 244 = 488 was the sample size we were aiming for at the second follow-up. Based on former experiences of the internet research agencies that we collaborated with in this study, a drop-out rate of 60% between the baseline and the second follow-up (T2) was estimated. Hence, we aimed to have data of 1220 participants available at the first measurement (after having applied various exclusion criteria) in order to have data of 488 participants available at the second follow-up.

### Procedure

Participants were recruited among members of two internet research agencies. Individuals were eligible to participate in the study when they were older than 18 years and had indicated earlier that they consume meat regularly. Participants who met the inclusion criteria, were invited by email. When willing to participate, they received the participants’ information explaining that the study aims to gain insight into the relationship between determinants related to eating behavior and that there would be three measurements (baseline, T1, T2). Further, they were informed that one measurement would take 15–20 min to complete, that each measurement entailed a reaction time task and a questionnaire, that no risks are related to the participation, that all data would be gathered and analyzed anonymously, and that they would receive a monetary reward for their participation. Depending on the standards for payment of the two different Internet panels, participants received €2.50 or €4.75 for participation in the baseline, €2.50 or €3.00 for participation in T1, and €4.00 or €5.00 for participation in T2. In order to begin with the study, an electronic informed consent needed to be read and agreed upon. If participants did not provide consent for participation, they were excluded from the study. In order to double-check whether only people who consumed red meat at least once a month participated, we included a question on this in the beginning. People who answered the question with ‘no’ were excluded from further participation. In the first part of the study, we assessed participants’ implicit attitudes towards red meat by means of a modified version of the Single-Category Implicit Association Test (SC-IAT) (Karpinski & Steinman, 2006). Afterwards, they filled in a questionnaire to measure explicit cognitions towards red meat consumption (explicit attitude comprised of perceived pros and perceived cons, social modeling, social norms, self-efficacy, intention to reduce red meat consumption) and red meat consumption. The participants had to perform the SC-IAT first, as a prior assessment of explicit cognitions might trigger red meat-related thoughts which would then in turn influence the reaction time in a following task (Bargh & Chartrand, 2000). One and three months after baseline, a new invitation was sent automatically to only those participants who had participated in the previous measure and were invited to complete the follow-up (e.g. T0 participants, who had not completed the T1 measurement, were not invited to participate at the T2 measurement). At both follow-ups, again participants’ implicit attitude towards red meat as well as all above-mentioned explicit cognitions and self-reported red meat consumption were assessed.

### Measurements

*Implicit attitude assessment task.* In order to assess implicit attitudes towards red meat, we used the SC-IAT for which satisfactory internal consistency has been demonstrated (Karpinski & Steinman, 2006). Since the IAT is based on comparisons between concepts, it always measures the association of attributes of one concept relative to another. As we were interested in the implicit attitudes towards red meat unrelated to an opposed category, the SC-IAT was chosen as it measures associations with a single category. As evaluative stimuli we used positive and negative words from the Affective Norms for English Words (ANEW) (Bradley & Lang, 1999) which were translated forth and back from English to Dutch by Dutch native researchers of Maastricht University. The Dutch words were then pretested regarding their perceived levels of valence (1 = ‘very negative’ to 9 = ‘very positive’), arousal (1 = ‘not arousing at all’ to 9 = ‘very arousing’), and familiarity (1 = ‘very unfamiliar’ to 9 = ‘very familiar’) by a sample of 28 people. Words with the highest scores regarding positivity and familiarity and similar arousal levels were selected as positive stimuli (*love*, *friend*, *freedom*, *humor*, *joy*; translated from Dutch). Words with the lowest scores on positivity, highest scores of familiarity and similar evaluations of arousal were selected as negative stimuli (*death*, *hate*, *devil*, *loneliness*, *lie*; translated from Dutch). To represent red meat, we selected pictures that were used in the study of De Houwer and De Bruycker (2007) and from the Internet which were free to be used (Creative Commons Images). These were pretested regarding their representativeness for red meat (1 = ‘not representative at all’, 2 = ‘not so strongly/a bit representative’, 3 = ‘strongly representative’). Based on this, seven pictures which were identified as the most representative for red meat were included in the SC-IAT.

The SC-IAT was programed by using the software Inquisit by Millisecond (Version 4) and the script was based on Karpinski and Steinman (2006). The SC-IAT contained two blocks which each consisted of 24 practice trials and 72 test trials. In one block ‘red meat or positive’ versus ‘negative’ built the two categories, in the reversed block ‘red meat or negative’ versus ‘positive’ were the two categories. One after one, pictures of red meat and negative or positive words appeared in the middle of the screen. Participants were instructed to indicate as rapidly as possible to which of the two categories the stimulus belonged. The two blocks were presented in a counterbalanced order, thus some participants received the block ‘red meat or positive’ versus ‘negative’ first and the reversed one subsequently whereas other had the block ‘red meat or negative’ versus ‘positive’ first and the reversed one afterwards. The idea underlying the SC-IAT is that when a person is quicker with categorizing the stimuli when ‘red meat or positive’ built one category than when ‘red meat or negative’ are one, the person’s implicit attitudes towards red meat is positive and vice versa. Throughout the task, category labels were displayed on the left and right upper part of the screen. When a presented stimulus belonged to the category displayed on the left upper part of the screen, participants had to press *e* on their keyboard. When the stimulus belonged to the category shown on the right upper part of the screen, they had to press *i*. All stimuli were presented in a randomized order and equally frequent. If an incorrect answer was given, a red X appeared on the screen until the answer was corrected.

The implicit attitude was indicated by d-scores. The d-score was calculated automatically by the Inquisit software using the D-algorithm proposed by Greenwald et al. (2003) with more positive values indicating a more positive reaction to red meat. D-scores can range from −2 to 2 and everyone in our sample scored between this range. After the SC-IAT, participants were asked whether they were distracted while performing the task, stating different types of distraction they could select (e.g. ‘I was talking on the phone’, ‘I was eating or drinking’, ‘I was listening to music’ etc.). Only when participants selected ‘I was not distracted’, their d-score was included in the analyses. To assess the internal reliability of the SC-IAT, the SC-IAT was divided into thirds (blocks of 24 test trials) and a SC-IAT score for each third was calculated (Karpinski & Steinman, 2006). The average intercorrelation among these scores was identified by means of the Spearman-Brown formula. This adjusted reliability coefficient is conceptually equivalent and directly comparable to Cronbach’s alpha. With a value of *r* = .73, the internal consistency was deemed acceptable.

*Self-report assessment.* The formulations of questions to measure explicit cognitions related to red meat intake were based on the I-Change model (De Vries, 2017; De Vries et al., 2005), which has previously been used to identify eating related cognitions (Schulz et al., 2014; Walthouwer, Oenema, Candel, Lechner, & de Vries, 2015). The questionnaire can be found at https://osf.io/7enj9/?view_only=d1afaf26fdbe4f13a9feb0d857c89db0.

Explicit attitude was assessed with two scales measuring the *perceived pros* and *perceived cons* of red meat consumption, which address underlying beliefs of the behavior. The content for the beliefs regarding meat consumption was derived from earlier studies (Dibb & Fitzpatrick, 2014; Verbeke & Viaene, 1999). *Perceived pros* and *perceived cons* were each expressed by 10 statements on a 5-point Likert Scale. One example for pros is ‘Eating red meat is’ (1) ‘not tasty’ to (5) ‘very tasty’. Due to low factor loadings, two perceived pros items were removed and a mean scale score was created of the remaining eight items and included in the analyses (Ω = .73). Higher values represent perceiving more pros. An example for cons is ‘Eating red meat is’ (1) ‘not unhealthy’ to (5) ‘very unhealthy’. One item had a low factor loading and was also removed. A mean scale score was created of the remaining nine items and included in the analyses (Ω = .66). A lower score represents perceiving fewer cons.

*Social norms* and *social modeling* were each assessed by four items. On a 5-point Likert scale, norms of family members, partners, and friends regarding reducing red meat consumption were assessed as well as their behavior. A norm item was ‘Most members of my family’ (1) ‘don’t think that I have to reduce my red meat intake’ to (5) ‘certainly think that I have to reduce my red meat intake’. A modeling item asked ‘How many of your family members consume red meat?’ with answers ranging from (1) ‘None of them’ to (5) ‘All of them’ or ‘My partner eats red meat’ with (1) ‘Yes’, (2) ‘No’, and (3) ‘I don’t have a partner/not applicable’ as answer options. We included a mean scale score for norms (Ω = .81) in the analyses and all four social modeling items were entered separately as latter construct showed a low internal structure (Ω = .20). Higher scores represent stronger norms or modeling.

*Self-efficacy* was assessed by nine items and was based on perceived barriers to reduce one’s meat intake (Dibb & Fitzpatrick, 2014). These items asked participants on a 5-point Likert scale to indicate to what extent they perceive themselves as capable of reducing their red meat intake, for instance ‘I will be able to reduce my red meat consumption even when I am used to eat red meat’ with answer options from (1) ‘completely disagree’ to (5) ‘completely agree’. A mean scale score was included in the analyses (Ω = .74). Higher scores indicate higher levels of self-efficacy.

*Intention* was measured by three items. The first item assessed whether respondents were planning to reduce their red meat intake, with answer options ranging from (1) ‘No, I am not planning to reduce my red meat intake’ to (7) ‘Yes, within one month’. The second item (likeliness to change) asked to indicate how likely it was that the person would reduce his/her red meat intake within the next three months, with answers from (1) ‘very unlikely’ to (5) ‘very likely’. The third item (intention strength) assessed how strongly the person was planning to reduce his/her red meat intake within the next three months. Answer options ranged from (1) ‘very little’ to (10) ‘very strongly’. Intention items were entered separately in the analyses as factor saturation of the standardized sum scores was estimated as insufficient (Ω = .07). Higher scores on all items represent a stronger intention.

Based on former diet-related studies (Springvloet, Lechner, Candel, De Vries, & Oenema, 2016; Van Assema, Brug, Ronda, Steenhuis, & Oenema, 2002) and the Food Frequency Questionnaire (FFQ), we assess *red meat consumption* by means of two items. Participants were asked on how many days per week they usually consume red meat (ranging from 1 to 7 days per week and the additional answer option ‘Not on a daily basis but at least once a month’) and how many grams they usually consume on these days (open question). To provide a reference point, we added the information that a piece of prepared meat at the main meal equals 100gr and a slice of meat topping (e.g. ham) equals 15gr. The weekly red meat consumption was calculated by multiplying the frequency by the amount of grams and was used in the analyses.

Further, we assessed participants age (‘What is your age’), sex (‘What is your gender?’) and level of education, which were used as cofounders in the analyses. Also we added two control questions (e.g. ‘From the following answer options, please select statement 4’) and excluded data of those participants who did not answer the control questions correctly.

### Analyses

To assess the scale quality of the measurements that were used in the present study, we first calculated their dimensionality by means of exploratory factor analyses. Subsequently, McDonald’s (2013) omega was calculated as a less biased alternative to Cronbach’s alpha (Crutzen & Peters, 2017). Compared to alpha, omega reduces the risks of under- and overestimation of internal consistency (Dunn, Baguley, & Brunsden, 2014) and has more realistic assumptions regarding variances of and covariance between items (Peters, 2014). Omega_hierarchical_ is based upon the sum of the squared loadings of items on the general factor. Values were calculated with R Studio and were presented in the measurements section above. All other statistical analyses were conducted using SPSS (IBM) version 24. Logistic regressions were used to investigate whether dropout at T1 and T2 was predicted by the variables age, gender, education, perceived pros, perceived cons, social modeling and social norms, self-efficacy, intention, and red meat consumption.

To answer the first research question, we performed three hierarchical multiple regressions, with the first regression having red meat consumption at baseline as dependent variable to investigate cross-sectional effects. In the second regression, we used red meat consumption after one month as dependent variable, and in the third one red meat consumption after three months as dependent variable in order to assess long-term effects. In all three regressions, baseline variables were added as predictors in three steps. In step 1, we entered age, gender, and education in step 2 perceived pros, perceived cons, social norms, social modeling, self-efficacy and intention, and in step 3 implicit attitudes as predictor.

To answer the second question, we added a fourth step to the abovementioned regressions, in which we entered all interaction terms between implicit attitude and the explicit cognitions. In case significant interaction terms were found, follow-up stratified analyses were conducted (Aiken, West, & Reno, 1991). In this case, implicit attitude was categorized into positive, neutral, and negative based on the tertiles of its score distribution. Implicit attitude scores ≤ −.167 were categorized as negative, scores > −.167 and ≤ .103 were considered neutral, and scores > .103 as positive implicit attitudes. In order to investigate whether the found interactions added significantly to the prediction of red meat consumption after one month or after three months, we performed another hierarchical regression analysis, only with the addition of the significant interaction terms.

To test the third and fourth questions, we performed hierarchical multiple regressions, similar to those carried out for question 2, but this time with intention each at baseline, after one month and after three months as dependent variable. In the first step, age, gender and education were entered; in the second step, baseline perceived pros, perceived cons, social norms, social modeling, and self-efficacy, implicit attitudes in a third step and in step 4, all interaction terms between implicit attitude and the explicit cognitions. All predictors were mean-centered before entering into the models.

## Results

### Descriptives

A total of 1790 individuals participated at baseline, out of which 314 were excluded as they either indicated to have been distracted during the SC-IAT or did not answer the control questions correctly. Hence a baseline sample of 1476 participants remained (47% female, mean age = 49, SD = 15.90). At the first follow-up after one month, 980 participants took part out of which 272 were excluded for the same reasons as mentioned above. Hence, the remaining sample at T1 consisted of 708 participants (48% of baseline, 47% female, mean age = 48, SD = 15.18). For the second follow-up, data of 556 participants were available out of which 89 were excluded. The remaining sample at T2 consisted of 467 data (32% of baseline, 44% female, mean age = 50, SD = 15.67). At follow-up one, having a partner who eats red meat predicted drop-out (T1: OR = 2.95, 95%CI [1.15, 7.53], *p* = .02). This variable was added in all analyses. No variable predicted drop-out at follow-up two. All characteristics of the sample as well as the differences of study variables over time are presented in Table 1. Correlations and 95% confidence intervals of the study variables at baseline are presented in Table 2. During the three waves, none of the participants reported to have ceased red-meat consumption. Red meat consumption was correlated with all measured study variables, except with social modeling (family members). Implicit attitudes were positively correlated with perceived pros and red meat consumption and negatively correlated with perceived cons, self-efficacy and all three intention items. Perceived pros and perceived cons were correlated to all other measured explicit cognitions.

**Table 1.** Characteristics of study sample and differences over time.

	T0 (*N* = 1476)	T1 (*n* = 708)	T2 (*n* = 467)	*F*	df	*p*
	M (SD)	M (SD)	M (SD)			
Age	49 (15.90)*	48 (15.18)	50 (15.67)**	1.22	2	.30
Gender (female), *n* (%)	692 (47%)	332 (47%)	178 (44%)	–	–	–
Perceived Pros	3.50 (.56)	3.54 (.59)	3.56 (.59)	1.92	2	.15
Perceived Cons	2.06 (.56)	2.08 (.56)	2.12 (.57)	2.57	2	.08
Social Norms	2.53 (.68)	2.48 (.70)	2.49 (.69)	1.65	2	.19
Social Modeling (partner, ‘Yes’), *n* (%)	992 (67)	475 (32)	329 (22)	–	–	–
Social Modeling (family members)	4.35 (.83)	4.36 (.79)	4.29 (.84)	1.17	2	.31
Social Modeling (friends)	3.96 (.70)	3.99 (.69)	3.92 (.71)	1.64	2	.20
Social Modeling (colleagues)	3.53 (.67)	3.56 (.65)	3.51 (.65)	1.18	2	.31
Self-efficacy	3.17 (.77)	3.18 (.77)	3.15 (.78)	.36	2	.70
Intention	2.24 (1.93)	2.30 (1.99)	2.40 (2.02)	1.26	2	.29
Intention (Likeliness to change)	2.08 (1.10)	2.03 (1.08)	2.11 (1.08)	.81	2	.45
Intention (Strength)	3.49 (2.53)	3.48 (2.53)	3.67 (2.54)	1.09	2	.34
Implicit attitude	−.03 (.32)	−.06 (.32)	−.05 (.31)	2.82	2	.06
Red meat consumption (gr/week)	473.50 (435.77)	493.06 (388.34)	484.23 (344.78)	.57	2	.57

**n = 1461, due to incomplete answers

**n = 401, due to incomplete answers

**Table 2.** Correlations (and 95%CI) between study variables at baseline.

Study variables	Correlations										
	1	2	3	4	5	6	7	8	9	10	11
1. Perceived Pros											
2. Perceived Cons	−.49** (−.53 to .45)										
3. Social Norms	−.23** (−.28 to .18)	.32** (.27-.37)									
4. Social Modeling (family members)	.28** (.23-.33)	−.25** (−.30 to .20)	−.29** (−.34 to .24)								
5. Social Modeling (friends)	.25** (.20–.30)	−.24** (−.29 to .19)	−.24** (−.29 to .19)	.41** (.36–.46)							
6. Social Modeling (colleagues)	.13** (.06–.20)	−.14** (−.21 to .07)	−.17** (−.22 to .12)	.32** (.26–.38)	.50** (.45–.55)						
7. Self-efficacy	−.33** (.08–.18)	.27** (−.19 to .09)	.09** (.04–.14)	−.09** (−.14 to .04)	−.07* (−.12 to .02)	−.05 (−.12 to .02)					
8. Intention	−.28** (−.33 to .23)	.46** (.42–.50)	.26** (.21–.31)	−.14** (−.19 to .09)	−.16** (−.21 to 11)	−.08* (−.15 to .01)	.22** (.17–.27)				
9. Intention (Likeliness to change)	−.36** (−.40 to .32)	.48** (.44–.52)	.29** (.24–.34)	−.19** (−.24 to .14)	−.20** (−.25 to 15)	−.11** (−.18 to .04)	.31** (.26–.36)	.71** (.68–.73)			
10. Intention (Strength)	−.33** (−.38 to .28)	.46** (.42–.50)	.29** (.24–.34)	−.22** (−.27 to .17)	−.22** (−.27 to .17)	−.15** (−.22 to .08)	.27** (.22–.32)	.70** (.67–.73)	.82** (.80–.84)		
11. Implicit attitude	.16** (.11–.21)	−.14** (−.19 to .09)	−.05* (−.01 to .001)	.04 (−.01 to .09)	.05 (−.01 to .10)	.04 (−.03 to .11)	−.10** (−.15 to .05)	−.06* (−.11 to .01)	−.08** (−.13 to .03)	−.07** (−.12 to .02)	
12. Red Meat Consumption	.32** (.27–.37)	−.20** (−.25 to .15)	−.06* (−.11 to .01)	.14** (.09–.19)	.17** (.12–.22)	.11** (.04–.18)	−.20** (−.25 to .15)	−.11** (.−16 to .06)	−.17** (−.22 to .12)	−.19** (.14–.24)	.09** (.04–.14)

*p <.05.

**p < .01.

Research question 1

*Do implicit attitudes and explicit cognitions predict (a reduced) red meat intake in addition to each other?*

Implicit attitudes did not add directly to the prediction of red meat consumption neither at baseline (*F*_change_ (1, 588) = .11, ^2^*β* = .01, *B* = 20.78, *p* = .74) nor after one month (*F*_change_ (1, 273) = .004, *β* = .004, *B* = 5.05, *p* = .95) or after three months’ follow-up (*F*_change_ (1, 161) = .53, *β* = -.05, *B* = −49.63, *p* = .47). At baseline, perceived pros (*β* = .20, *B* = 182.88, *p* < .001, 95%CI^3^ [97.53, 268.24]) and intention strength (*β* = −.15, *p* = .04 *B* = −28.52, 95%CI [−55.53, −1.51]) were significant predictors for red meat consumption, explaining 12% of variance.

After one month, perceived pros (*β* = .28, *B* = 255.08, *p* < .001, 95%CI [120.91, 329.25]), norms (*β* = .12, *B* = 64.06, *p* = .05, 95%CI [.68, 127.44]) and intention to change (*β* = .16, *B* = 36.68, *p* = .05, 95%CI [.56, 72.81]) explained 21% of variance in red meat consumption and after three months, being male (*β* = −.15, *B* = −99.93, *p* = .04, 95%CI [−193.94, −5.92]), perceived pros (*β* = .36, *B* = 214.79, *p* < .001, 95%CI [116.77, 312.81]) and intention strength (*β* = −.30, *B* = −42.49, *p* = .04, 95%CI [−82.09, −2.90]) explained 31% of variance in red meat consumption.

Research question 2

*Do implicit attitudes and explicit cognitions interact in the prediction of (a reduced) red meat consumption?*

At baseline, the interaction between implicit attitudes and self-efficacy showed a trend towards significance (*β* = .08, *B* = 150.89, *p* = .08, 95%CI [−16.31, 318.09]). Follow-up stratified analyses demonstrated that the effect was strengthened significantly by negative implicit attitudes towards red meat (*β* = −.20, *B* = −83.92, *p* = .006, 95%CI [−142.95, −24.89]) but not for neutral (*β* = −.02, *B* = −15.12, *p* = .83, 95%CI [−156.90, 126.66]) or positive implicit attitudes towards red meat (*β* = .06, *B* = 28.72, *p* = .42, 95%CI [−41.08, 98.51]). This indicates that the effect of self-efficacy on red meat consumption is strengthened when the person holds a negative implicit attitude towards red meat. Along with perceived pros and intention strength, the interaction between self-efficacy and implicit attitude added significantly to the prediction of red meat consumption (*F*_change_ (1, 588) = 4.93, *β* = .09, *B* = 173.88, *p* = .03) and explained 13% of the variance. After one and after three months, no significant interaction effects between implicit attitudes and explicit cognitions were detected.

Research question 3

*Do implicit attitudes and explicit cognitions interact in the prediction of the intention to reduce red meat consumption?*

At baseline the interaction between social norms and implicit attitudes showed a trend towards significance regarding intention (item 1) (*β* = .07, *B* = .54, *p* = .07, 95%CI [−.04, 1.11]). Stratified analyses demonstrated that this effect was significant when implicit attitudes were negative (*β* = .15, *B* = .38, *p* = .03, 95%CI [.03, .73]) and positive (*β* = .28, *B* = .62, *p* < .001, 95%CI [.31, .92]) but not when they were neutral (*β* = .10, *B* = .23, *p* = .15, 95%CI [−.08, .54]). This indicates that the effect of social norms on red meat consumption is strengthened when the individual holds a negative or a positive implicit attitude towards red meat. Regarding the items intention likeliness and intention strength, no significant interactions were found.

After one month’ follow-up, interactions were non-significant. After three months, only the interaction between implicit attitudes and social norms regarding the intention likeliness to change was significant (*β* = −.21, *B* = −.69, *p* = .03, 95%CI [−1.31, −.08]). Stratified analyses, however, did not reveal significant results (negative: *β* = .10, *B* = .12, *p* = .46, 95%CI [−.20, .43]; neutral: *β* = .06, *B* = .09, *p* = .68, 95%CI [−.35, .53]; positive: *β* = .02, *B* = .03, *p* = .89, 95%CI [−.37, .43]).

Research question 4

*Do implicit attitudes and explicit cognitions predict the intention to reduce red meat consumption in addition to each other?*

At baseline, no direct effects of implicit attitudes were detected regarding the intention to reduce red meat intake (item 1) (*F*_change_ (1, 591) = .05, *β* = −.01, *B* = −.05, *p* = .83), intention likeliness (*F*_change_ (1, 591) = .88, *β* = −.03 *B* = −.11, *p* = .35), and intention strength (*F*_change_ (1, 591) = .002, *β* = .002, *p* = .96). Education (*β* = .08, *B* = .13, *p* = .03, 95%CI [.02, .24]), perceived cons (*β* = .37, *B* = 1.28, *p* <.001, 95%CI [.98, 1.58]), social norms (*β* = .18, *B* = .43, *p* < .001, 95%CI [.24, .61]), having a partner who does not eat red meat (*β* = −.10, *B* = −.96, *p* = .007, 95%CI [−1.65, −.27]) and self-efficacy (*β* = .12, *B* = .33, *p* = .001, 95%CI [.14, .51]) explained 30% of the intention (item 1). Intention likeliness was explained by being female (*β* = .10, *B* = .23, *p* = .005, 95%CI [.07, .38)], age (*β* = .07, *B* = .006, *p* = .05, 95%CI [.00, .01]), perceived pros (*β* = −.12, *B* = −.24, *p* = .004, 95%CI [−.41, −.08]), perceived cons (*β* = .31, *B* = .60, *p* < .001, 95%CI [.44, .77]), social norms (*β* = .19, *B* = .26, *p* < .001, 95%CI [.16, .36]), and self-efficacy (*β* = .19, *B* = .29, *p* < .001, 95%CI [.19, .39]) which together explained 37% of the variance. Intention strength was predicted by being female (*β* = .09, *B* = .43, *p* = .02, 95%CI [.07, .80]), age (*β* = .13, *B* = .02, *p* = .001, 95%CI [.01, .04]), perceived cons (*β* = .34, *B* = 1.46, *p* < .001, 95%CI [1.09, 1.84]), social norms (*β* = .17, *B* = .51, *p* < .001, 95%CI [.28, .73]), having a partner who does not eat red meat (*β* = −.07, *B* = −.87, *p* = .05, 95%CI [−1.73, −.02]), the amount of friends eating red meat (*β* = −.10, *B* = −.38, *p* = .02, 95%CI [−.70, −.05]), and self-efficacy (*β* = .14, *B* = .45, *p* < .001, 95%CI [.22, .68]) which together explained 33% of variance.

After one month, implicit attitudes added directly to intention (item 1) (*F*_change_ (1, 276) = 5.52, *β* = −.12, *B* = −.83, *p* = .02) and explained along with perceived cons (*β* = .43, *B* = 1.54, *p* < .001, 95%CI [1.09, 1.98]) and norms (*β* = .19, *B* = .49, *p* = .001, 95%CI [.21, .76]) 29% of the variance. Also regarding the item intention likeliness, implicit attitudes added significantly to the prediction (*F*_change_ (1, 276) = 4.45, *β* = −.10, *B* = −.38, *p* = .04). Together with age (*β* = .13, *B* = .01, *p* = .02, 95%CI [.002, .02]) and the explicit cognitions perceived cons (*β* = .47, *B* = .89, *p* < .001, 95%CI [.66, 1.12]) and norms (*β* = .17, *B* = .23, *p* = .001, 95%CI [.09, .38]), they explained 36% of the variance. Intention strength was not directly explained by implicit attitudes (*F*_change_ (1, 276) = 2.71, *β* = −.08, *B* = −.67, *p* = .10) but was predicted by age (*β* = .12, *B* = .02, *p* = .03, 95%CI [.003, .04]), perceived cons (*β* = .49, *B* = 2.09, *p* < .001, 95%CI [1.58, 2.61]), and norms (*β* = .16, *B* = .48, *p* = .003, 95%CI [.16, .80]). All regression coefficients are depicted in Table 3.

**Table 3.** Coefficients of the hierarchical multiple regression analysis with intention, intention likeliness, and intention strength at T1 as dependent variables. Implicit attitude is added in step 3.

Block	Independent variables	Intention at T1					Intention Likeliness at T1					Intention Strength at T1				
		B	SE	*β*	95%CI	*p*	B	SE	*β*	95%CI	*p*	B	SE	*β*	95%CI	*p*
1	Gender	0.16	0.25	0.04	−0.33 to 0.65	0.51	0.23	0.13	0.10	−0.04 to 0.49	0.09	0.60	0.30	0.12	0.01–1.19	0.05
	Age	0.01	0.01	0.06	−0.01 to 0.03	0.33	0.01	0.01	0.09	−0.003 to 0.02	0.14	0.02	0.01	0.08	−0.01 to 0.04	0.18
	Education	0.29	0.10	0.18	0.09 to 0.49	0.004	0.13	0.05	0.15	0.02 to 0.24	0.02	0.25	0.12	0.13	0.01 to 0.49	0.04
2	Gender	−0.21	0.23	−0.05	−0.66 to 0.25	0.37	−0.05	0.12	−0.02	−0.28 to 0.18	0.65	−0.02	0.26	−0.004	−0.54 to 0.50	0.94
	Age	0.02	0.01	0.11	0.00–0.03	0.05	0.01	0.00	0.13	0.00–0.02	0.02	0.02	0.01	0.12	0.003–0.04	0.03
	Education	0.13	0.09	0.08	−0.05 to 0.31	0.16	0.04	0.05	0.05	−0.05 to 0.13	0.38	0.06	0.11	0.03	−0.14 to 0.27	0.55
	Perceived Pros	0.20	0.24	0.05	−0.27 to 0.67	0.40	−0.10	0.12	−0.05	−0.34 to 0.14	0.40	−0.02	0.27	−0.004	−0.55 to 0.52	0.94
	Perceived Cons	1.53	0.23	0.43	1.08–1.98	<.001	0.89	0.12	0.47	0.66–1.12	<.001	2.10	0.26	0.49	1.58–2.61	<.001
	Social Norms	0.50	0.14	0.20	0.22–0.78	0.001	0.24	0.07	0.18	0.09–0.38	0.001	0.48	0.16	0.16	0.16–0.80	0.001
	Social Modeling (partner)	0.36	0.71	0.03	−1.04 to 1.76	0.61	0.59	0.36	0.08	−0.12 to 1.30	0.10	1.16	0.81	0.07	−0.44 to 2.76	0.16
	Social Modeling (family members)	0.31	0.18	0.10	−0.05 to 0.67	0.10	0.05	0.09	0.03	−0.13 to 0.24	0.56	0.24	0.21	0.07	−0.17 to 0.65	0.25
	Social Modeling (friends)	−0.07	0.22	−0.02	−0.50 to 0.36	0.75	−0.06	0.11	−0.04	−0.28 to 0.15	0.56	−0.29	0.25	−0.07	−0.78 to 0.20	0.24
	Social Modeling (colleagues)	0.07	0.19	0.02	−0.29 to 0.44	0.69	0.10	0.10	0.06	−0.09 to 0.28	0.32	0.03	0.21	0.01	−0.39 to 0.45	0.91
	Self-efficacy	0.20	0.15	0.07	−0.10 to 0.49	0.19	0.11	0.08	0.07	−0.04 to 0.26	0.15	0.11	0.17	0.03	−0.23 to 0.45	0.54
3	Gender	−0.23	0.23	−0.06	−0.67 to 0.22	0.32	−0.06	0.12	−0.03	−0.29 to 0.17	0.60	−0.04	0.26	−0.01	−0.55 to 0.48	0.89
	Age	0.02	0.01	0.11	−0.001 to 0.03	0.06	0.01	0.004	0.13	0.002 to 0.02	0.02	0.02	0.01	0.12	0.002 to 0.04	0.03
	Education	0.13	0.09	0.08	−0.05 to 0.31	0.16	0.04	0.05	0.05	−0.05 to 0.13	0.38	0.06	0.11	0.03	−0.14 to 0.27	0.55
	Perceived Pros	0.29	0.24	0.07	−0.18 to 0.75	0.23	−0.06	0.12	−0.03	−0.30 to 0.18	0.60	0.05	0.27	0.01	−0.49 to 0.59	0.86
	Perceived Cons	1.54	0.23	0.43	1.09–1.98	<.001	0.89	0.12	0.47	0.66–1.12	<.001	2.10	0.26	0.49	1.59–2.62	<.001
	Social Norms	0.49	0.14	0.19	0.21–0.76	0.001	0.23	0.07	0.17	0.09–0.37	0.002	0.47	0.16	0.16	0.15–0.79	0.004
	Social Modeling (partner)	0.29	0.71	0.02	−1.10 to 1.67	0.68	0.56	0.36	0.08	−0.15 to 1.27	0.12	1.10	0.81	0.07	−0.49 to 2.70	0.18
	Social Modeling (family members)	0.30	0.18	0.10	−0.06 to 0.65	0.10	0.05	0.09	0.03	−0.13 to 0.23	0.59	0.23	0.21	0.07	−0.18 to 0.64	0.27
	Social Modeling (friends)	−0.06	0.22	−0.02	−0.49 to 0.37	0.78	−0.06	0.11	−0.03	−0.28 to 0.16	0.59	−0.28	0.25	−0.07	−0.77 to 0.21	0.26
	Social Modeling (colleagues)	0.09	0.19	0.03	−0.27 to 0.45	0.63	0.10	0.09	0.06	−0.08 to 0.29	0.28	0.04	0.21	0.01	−0.38 to 0.46	0.86
	Self-efficacy	0.19	0.15	0.07	−0.11 to 0.48	0.21	0.11	0.08	0.07	−0.04 to 0.26	0.16	0.10	0.17	0.03	−0.24 to 0.44	0.56
	Implicit attitude	−0.83	0.35	−0.12	−1.52 to 0.13	0.02	−0.38	0.18	−0.10	−0.73 to 0.03	0.04	−0.67	0.41	−0.08	−1.47 to 0.13	0.10

Note*.* B = unstandardised regression coefficient; *β* = standardised regression coefficient.

After three months, intention (item 1) was not explained by implicit attitudes (*F*_change_ (1, 164) = .31, *β* = −.0 4, *B* = −.24, *p* = .58) but by perceived cons only (*β* = .40, *B* = 1.49, *p* < .001, 95%CI [.82, 2.17]). Also the items intention likeliness and intention strength were predicted by perceived cons only (intention likeliness: *β* = .37, *B* = .67, *p* < .001, 95%CI [.33, 1.02]; intention strength: *β* = .43, *B* = 1.92, *p* < .001, 95%CI [1.13, 2.70]) and not by implicit attitudes (intention likeliness: *F*_change_ (1, 164) = .41, *β* = .05, *B* = .14, *p* = .52; intention strength: *F*_change_ (1, 164) = .03, *β* = −.01, *B* = −.08, *p* = .87).

## Discussion

The study at hand provided insight into how implicit attitudes and explicit cognitions operate in the prediction of the intention to reduce red meat intake as well as in the prediction of (a reduced) red meat consumption. Additive as well as interaction patterns between these determinants were examined.

Implicit attitudes were found to be weakly positively correlated with red meat consumption (at baseline), but were not associated with red meat consumption after controlling for explicit cognitions neither at baseline nor at any later measuring point. Thereby, our results do not suggest support for the existence of the additive pattern for the prediction of red meat consumption. To our knowledge, this is the first study to investigate the direct effect of both implicit attitudes and explicit cognitions on red meat consumption. Only one similar study in the context of food choices has been conducted by Richetin et al. (2007) who found implicit attitudes to predict a person’s snack and food choice besides explicit attitudes, thus evidence for the additive pattern. This pattern was not found for red meat consumption allowing to conclude that the mode of operation between implicit and explicit determinants is not generalizable to one domain (e.g. eating behavior) but differs depending on the specific (eating) behavior. Another explanation for the different findings could be the different (methodological) approaches. Whereas Richetin et al. (2007) used the IAT and confirmed the additive pattern for the choice of snacks relative to the choice of fruits, we assessed the implicit attitude towards red meat unrelated to an opposed category. Further, we used a self-report measurement in order to assess red meat consumption. Although this was based on the Food frequency scale – a widespread and accepted way of measuring food intake – self-reports are prone to reporting errors. Richetin et al. (2007) on the contrary used more direct assessments, i.e. participants had to choose a snack or a fruit after the experiment. Thus potential self-report bias was excluded. However, it needs to be stressed that the behavioral measure of Richetin et al. (2007) was conducted directly after the experiment, and therefore priming or social desirability effects that occurred during the experiment cannot be ruled out. In order to draw a more generalizable conclusion about whether red meat consumption is directly influenced by implicit attitudes or not, a follow-up study with a more objective measure of red meat consumption is recommended.

Although the additive pattern could not be supported, support for the interactive pattern was found. At baseline, the relationship between self-efficacy and red meat consumption was moderated by implicit attitudes and significantly strengthened by negative implicit attitudes. That is, people who consider themselves (explicitly) as capable of reducing their red meat intake, show a lower red meat consumption especially when they hold a negative implicit attitude towards red meat. This appears logical and is in line with findings of a study from Muschalik et al. (2018) who also found implicit attitudes to moderate the relationship between self-efficacy and physical activity behavior. This finding supports the idea of an interactive pattern of influencing red meat consumption and suggests that negative implicit attitudes are beneficial in order to foster the likelihood that self-efficacy decreases red meat intake in the short-term.

Regarding the intention to reduce one’s red meat intake, support for the additive as well as for the interactive pattern of operation was found. This is in line with our expectation that both ways of operation do not exclude each other. More precisely, baseline implicit attitudes significantly predicted the intention to change one’s red meat intake as well as the intention likeliness after one month, with more positive implicit attitudes towards red meat being associated with a lower intention. Although a few studies have considered direct effects of implicit processes on intention (Brochu & Morrison, 2007; Cin et al., 2007; Dasgupta & Rivera, 2008), this is still a rather uncommon approach as the effect of implicit attitudes is mostly investigated on behavior. The findings of our studies, which are in line with former studies, indicate however that implicit (and explicit) attitudes are not exclusively associated with behavior but also with its’ most proximate determinant intention. Based on our findings and the findings of other authors, we suggest a reconsideration of the assumptions made in theoretical models, i.e. RIM (Strack & Deutsch, 2004), which mostly assume only a direct effect of implicit attitudes on behavior but not on intention. Hence, adding intention in the RIM appears to be a logical extension.

Furthermore, not only a direct but also an indirect effect of implicit attitudes on intention was identified. That is, the interaction between social norms and implicit attitudes was significant for baseline intention to change and showed a curvilinear relationship. Hence, the effect of social norms on the intention to change was strengthened by positive as well as by negative implicit attitudes towards social norms, but not by neutral implicit attitudes. The finding that negative implicit attitudes strengthen the effect of social norms on intention appears logical, as it implies that other peoples’ expectations to reduce one’s red meat consumption have a stronger increasing effect on intention when the person also has a negative implicit attitude towards red meat. The very same relationship was also strengthened by positive implicit attitudes and seems surprising in the first place. However, Muschalik et al. (2018) found a similar pattern. In their study, the effect of social modeling on the intention to become physically active was significantly strengthened by negative implicit attitudes towards physical activity. The authors argued that resulting from the opposing cognitions, participants experienced dissonance and were motivated to resolve this dissonance that is normally associated with tensed or negative feelings. In order to do so, they assumingly denied the negative implicit attitude and acted in line with the (more accessible) behavior of others. It is plausible that also in the present study, the explicit knowledge that other people expect oneself to reduce ones’ red meat consumption and the opposing unconscious preference for red meat created dissonance. In this case, people would also be motivated to resolve this dissonance and one way to do so could be by denying the less accessible positive implicit attitude and by acting in accordance with the more available social norms – i.e. indicating a high intention to change. Although this way of dissonance resolving has not been demonstrated in the context of eating behaviors, a similar technique was revealed in a study about smoking (Maliszewski, 2011) in which people with a negative implicit attitude and a positive explicit attitude towards smoking resolved this conflict by inhibiting their less accessible negative implicit attitude and by acting upon the more accessible positive explicit attitude, i.e. by smoking a cigarette. It is conceivable, that participants in the present study used a similar approach as it appears easier to follow the more obvious and accessible norms of others than the unconscious attitude. Although one could argue that positive implicit attitudes towards red meat were beneficial in this context, one has to take into account that this was assumingly induced by dissonance. In case that social norms are low (e.g. an individual does not experience other people to expect him or her to reduce red meat intake), a positive implicit attitude towards red meat would possibly strengthen this effect and lower the individual’s intention.

Based on our findings, it can be said that next to tackling explicit cognitions, health interventions that are aiming at an intake below 300gr/week could benefit from training or changing implicit attitudes regarding red meat towards a negative direction. Until now, this is not a common approach as interventions to reduce meat consumption mostly address explicit cognitions, e.g. by means of self-monitoring (Carfora, Caso, & Conner, 2017a; Carfora, Caso, & Conner, 2017b). Although these studies led to a lower meat intake, one could argue that changing implicit attitudes in addition to that might increase effectiveness of such interventions, as implicit attitudes were also related to reduced red meat intake and intention in our study. An attempt to alter food related implicit attitudes into a negative direction has been undertaken by Hollands, Prestwich, and Marteau (2011). They paired images of energy-dense snack foods with aversive images of the potential health consequences of unhealthy eating and found baseline positive implicit attitudes towards energy-dense snacks to be more negative after the experiment. Although this approach has not been applied to the consumption of red meat, pairing pictures of red meat with aversive images in a computerized task appears to be a feasible way to complement interventions that already aim at altering explicit cognitions towards red meat consumption. Additionally, our results suggest that the role and place of implicit associations may not always be completely distinct as suggested by the RIM (Strack & Deutsch, 2004). Social cognitive models, such as the Reasoned Action Approach (Fishbein & Ajzen, 2011) or the I-Change Model (De Vries, 2017), need to address the importance of implicit associations and research is needed to identify how to best depict the pathways of both implicit and explicit factors. Moreover, newer studies suggest the existence of single-process models of attitudes rather than the existence of dual-process models, which was the point of departure in the present study. Single-process models state that it is debatable whether there rightly exists the distinction of a reflective and impulsive system (e.g. Hu, Gawronski, & Balas, 2017; Moran & Bar-Anan, 2013). In order to draw more generalizable conclusions about the correct depiction of attitude models, future studies regarding this topic are encouraged.

### Limitations

When interpreting our findings, the following possible limitations need to be taken into account. First, as mentioned above, we assessed participants’ red meat consumption by means of self-reports which is prone to reporting errors. In order to confirm that the additive pattern cannot be applied to red meat consumption, a follow-up study with a measure of red meat intake that is less sensitive to bias would be valuable (e.g. a food diary, pictures of consumed food). Furthermore, the intention constructs were entered separately into the analyses. Although the use of single-item scales is a widespread and accepted method to assess (dietary related) intention (Ajzen & Fishbein, 1980; Grønhøj, Bech-Larsen, Chan, & Tsang, 2012; Øygard & Rise, 1996; Patch, Tapsell, & Williams, 2005; Rezai, Teng, Mohamed, & Shamsudin, 2012), constructs measured by several items or a full scale are agreed on to be more reliable (Lowenthal, 2001; Nunnally & Bernstein, 1994). Therefore, considering a follow-up study with multi-item measures of the intention constructs would be worthwhile. In addition, Hofmann, Gschwendner, Nosek, and Schmitt (2005) argued that the relation between implicit and explicit attitudes can be influenced by design factors, such as the correspondence of measurements. We assessed explicit cognitions (including explicit attitude) regarding red meat consumption and implicit attitudes towards red meat, as implicit attitudes towards red meat consumption appeared to be rather difficult to be assessed via the SC-IAT, i.e. stimuli that clearly represents the consumption of red meat. This is a general problem of the SC-IAT. Many other studies used a similar approach, e.g. assessing implicit attitudes towards smoking by using smoking related stimuli (e.g. words/pictures of cigarettes, tobacco, nicotine, ashtray etc.) to predict smoking behavior (Huijding, de Jong, Wiers, & Verkooijen, 2005; Waters et al., 2007). However, as a consequence, the correspondence between the measurements might have been reduced. Not only follow-up studies should take this into account, but more research regarding the design of the SC-IAT is needed in order to find a possible solution for this issue.

## Conclusion

This research has taken an important first step to illustrate that both explicit cognitions and implicit attitudes are associated with the intention to reduce ones’ red meat intake as well as with red meat consumption and demonstrated that negative implicit attitudes towards red meat are more beneficial when aiming at a higher intention and a lower red meat intake. Future research should seek to replicate the findings of additive and interactive patterns and should also examine whether tackling both implicit attitudes and explicit cognitions does indeed result in more significant decreases of red meat intake than when tackling one type of cognition only. Shedding light on these questions may help to achieve a transition to a less meat-based diet and could thereby improve peoples’ health as well as the health of the planet.

## Data Availability

Materials used in this study as well as non-identifiable data, syntax, and output of the analyses are available at https://osf.io/7enj9/.

## Notes

1 In the literature, there exists no common agreement about whether implicit associations are rightly defined as unconscious in the sense that people are not aware of them. Other authors prefer, therefore the terms ‘direct’ and ‘indirect’ measures. However, since at least certain aspects of implicit associations are defined as unconscious, we use the terms unconscious and conscious throughout the manuscript.

2 *β* = standardized beta; *B* = unstandardized beta

3 Unstandardized 95% CIs are reported in the whole manuscript.
